# Influential Plan to Spread Knowledge

**Published:** 1920-01-31

**Authors:** 


### LEAGUE OF HEALTH.

### Influential Plan to Spread Knowledge.

The inaugural meeting of the People's League of Health, of which Miss Olga Nethersole is the founder and organiser, was a complete success. It has the support of a Council of leading medical men and an Executive Committee of welfare workers. Its objects are social research, the improvement of conditions of living as they affect health, and the spread of knowledge. A national campaign has been planned, and so sound a project deserves success.

Lord Burnham, who took the chair, made a valuable point in his statement—undoubtedly true—that the statistics of mortality, disease, and defective men, women, and children would be radically reduced if the public had knowledge of the powers which they possess under various Acts of Parliament. It is lamentable, he said, that the powers which now exist, should have been so inadequately carried into effect, and if the many eminent medical men on the Council were properly supported by the driving force of public opinion much might be achieved.

Miss Nethersole explained that her determination to start the League was formed as the result of her experiences when, during the war, she helped to compile the national register. In the part of Southwark which was assigned to her she was ashamed to find conditions so insanitary and unhygienic. As one instance of the need of such an organisation, she said she had it on the authority of Sir George Newman, Chief Medical Officer of the Ministry of Health, that a million children were so defective, mentally and physically, as to be unable to derive reasonable benefit from the education provided for them. This is a double waste. Miss Nethersole was also on firm ground when she pleaded for a chance to be given to medical scientists—as a change from the lawyers—to improve national conditions. Medical experts are not influenced by political considerations. Another important point which Miss Nethersole emphasised was the employment of children, over which she urged the strictest supervision. The League is an excellent conception, influentially backed by brains, and its progress will be watched with interest and hope.

Members of the League are to receive lectures from members of the medical profession, and public exhibitions of a popular rather than scientific nature are to be arranged at all large centres throughout the Empire. The subjects dealt with at such exhibitions are exceedingly numerous, and include :

Tuberculosis.—Showing all the latest methods of prevention and cure of tuberculosis, at which a special series of lectures shall be given to the general public on "The Sociological Importance of Tuberculosis," "Tuberculosis in the School," "Tuberculosis in the Factory and the Shops," and "The Municipal Control of Tuberculosis." Food.—With addresses on its properties and value to the human body, exhibiting a new "People's Cookery Book," compiled for health and not for palate, by medical authorities. Housing.—The value of the suburb to the workers and their children. Waste.—Human and otherwise. Teeth.—The results of bad teeth and their relation to oral sepsis and ill-health.

Alcohol.—Its value and abuse. The food value of beer when containing not more than 2 per cent. of alcohol and when taken in moderation.

Physical Education and Gymnastics.—Out-of-door exercise and recreation—to preserve mental and physical health.

Lord Burnham, in opening the first meeting of the League, said that their object was directed entirely to a propaganda of public welfare, of love of things which were good, and prominent among these was health. The League would begin by fighting public ignorance which was at the root of our troubles. It was doubtful whether so many eminent medical men had ever been included in any body outside their own professional organisations as were to be found on the Medical Council of the League. It only remained for the public to support those experts.

Sir Alfred Fripp emphasised the fact that there was no intention to rival the activities of existing agencies, or to act in opposition to the Government. The idea of the League was to centralise and co-ordinate all effort, and he suggested that the present offered a rare opportunity of educating the people in matters of health, for the public mind was in a receptive state.

Many of the objects of this League are not new, and some of them are somewhat Utopian, but we feel that nothing but good can accrue from this new organisation to fight disease, and we wish it every success.

